# Role of the nucleotide excision repair endonuclease XPF in the kinetoplastid parasite *Trypanosoma brucei*

**DOI:** 10.1038/s41598-025-08659-y

**Published:** 2025-07-02

**Authors:** Claudia Gómez-Liñán, María Sáez-Maldonado, Laura Montosa-Hidalgo, Luis Miguel Ruiz-Pérez, Dolores González-Pacanowska, Antonio E. Vidal

**Affiliations:** Instituto de Parasitología y Biomedicina López-Neyra (IPBLN), Consejo Superior de Investigaciones Científicas (CSIC), Parque Tecnológico de Ciencias de la Salud, Avenida del Conocimiento, 17, Armilla, Granada, 18016 Spain

**Keywords:** *Trypanosoma brucei*, Nucleotide excision repair, NER, XPF, ERCC4, Parasitology, Nucleotide excision repair

## Abstract

The nucleotide excision repair (NER) mechanism is responsible for removing bulky DNA damage, such as pyrimidine dimers induced by ultraviolet (UV) light. The NER pathway excises the damaged strand through incisions at the 5’ and 3’ ends of the damage, with the 5’ incision catalyzed by the XPF-ERCC1 endonuclease complex. Here, we identify an XPF ortholog in *Trypanosoma brucei*, the causative agent of human African trypanosomiasis (sleeping sickness). XPF-deficient parasites exhibit hypersensitivity to UV irradiation and a slower rate of DNA damage repair. Consistent with its role in DNA repair, XPF localizes to the nucleus, associating with nucleoplasmic and nucleolar regions. Additionally, we demonstrate that TbXPF protects against intra- and inter-strand crosslinks induced by cisplatin and mitomycin C, respectively. The presence of a functional NER pathway in trypanosomes suggests that these organisms are susceptible to replication- and transcription-blocking DNA damage *in vivo*. Under genotoxic stress, genome stability and parasite survival may heavily rely on DNA repair systems such as NER which, for this reason, could be an effective target for chemotherapeutic interventions.

**Supplementary Information:**

The online version contains supplementary material available at 10.1038/s41598-025-08659-y.

## Introduction

Nucleotide excision repair (NER) is a versatile DNA repair system responsible for removing diverse helix-distorting bulky lesions, such as those formed by UV light, environmental mutagens, and certain cancer chemotherapeutic adducts. In humans, defective NER leads to several autosomal recessive disorders, including xeroderma pigmentosum (XP), Cockayne syndrome (CS), UV-sensitive syndrome (UVSS), cerebro-oculo-facio-skeletal syndrome (COFS), and trichothiodystrophy (TTD)^1^. The mammalian NER pathway utilizes more than 30 proteins to detect DNA lesions, excise approximately 24–32 nucleotides from the damaged strand, fill the gap using the undamaged strand as a template, and complete the error-free repair by sealing the strand^2^. Within NER, two damage detection mechanisms exist: global genome repair (GG-NER) that eliminates DNA lesions throughout the genome, and transcription-coupled repair (TC-NER) that specifically repairs damage from the template DNA strands of actively transcribed genes. While GG-NER is initiated by the recognition of DNA helix-distorting damage, a blocked RNA polymerase II (RNA Pol II) at a lesion constitutes the first step for damage recognition in TC-NER. The XPC complex formed by XPC, RAD23B and centrin 2 and the UV-DDB (ultraviolet radiation-DNA damage-binding protein) complex, consisting of DDB1 and DDB2, constitute the main DNA damage sensors in GG-NER. In TC-NER, lesion-stalled RNA Pol II recruits the TC-NER-specific factors Cockayne syndrome proteins CSA and CSB, required for further assembly of the NER machinery. Once the lesion is detected, both sub-pathways converge into the same route. First, DNA damage is verified by TFIIH (transcription initiation factor IIH), which is a transcription initiation and repair complex containing ten protein subunits including XPB and XPD helicases. The existence of a DNA lesion is further verified by the XPA protein, which binds to single-stranded, chemically altered nucleotides. The next step is DNA damage excision, catalyzed by the structure-specific endonucleases XPF-ERCC1 (recruited by XPA) and XPG, which incise the damaged strand at 5’ and 3’ ends of the lesion, respectively. Gap-filling DNA synthesis starts right after the 5’ incision and involves PCNA, RFC, RPA and distinct DNA polymerases and ligases, depending on the cell’s proliferation status. In replicating cells, DNA Pol ε performs DNA synthesis, and DNA ligase 1 seals the strand, whereas in non-replicating cells, DNA Pol δ, DNA Pol κ, and the XRCC1-DNA ligase 3 complex take over this process (reviewed in^2,3^).

Both XPF (ERCC4; excision repair cross-complementing group 4 protein) and ERCC1 belong to the XPF/MUS81 family^4^. They form a stable heterodimer composed of a catalytic (XPF) and a non-catalytic (ERCC1) subunit. The XPF-ERCC1 complex (Rad1-Rad10 in yeast) cleaves DNA 5′ to UV-induced lesions by recognizing 3′ single-stranded DNA at a double-strand/single-strand DNA junction^5^. XPF-ERCC1 also plays roles beyond NER, being involved in DNA double-strand break repair, base excision repair, DNA inter-strand crosslink repair, and telomere maintenance^6–9^. The multifunctional role of the complex XPF-ERCC1 underlies the remarkable array of disorders found in human patients and mouse models defective in XPF or ERCC1. Thus, mutations in the XPF and ERCC1 genes have been associated with various clinical phenotypes, including abnormal skin photosensitivity, late-onset skin cancers, neurological diseases, and accelerated aging in both human patients and mice^10^.

The unicellular protozoan parasite *Trypanosoma brucei* is the causative agent of African trypanosomiasis (HAT) or sleeping sickness in humans and nagana disease in cattle. These parasites are transmitted through the bite of a tsetse fly (*Glossina* spp.). Once in the mammalian host, trypanosomes replicate extracellularly in the bloodstream, then cross the blood–brain barrier to initiate the second stage of the disease, causing the characteristic sleep disorder^11^. HAT affects millions of people throughout sub-Saharan Africa and is usually fatal if untreated or inadequately treated. According to the World Health Organization, HAT remains one of the most significant and challenging neglected tropical diseases to eradicate due, among other factors, to the need of new therapeutic strategies^12^.

Many DNA damage repair pathways are conserved across eukaryotes, although variations and specializations exist among different organisms. For example, trypanosomatids lack the canonical non-homologous end joining (NHEJ) pathway for double-strand break repair^13^. Many of the GG-NER and TC-NER components identified in mammals have also been found in *T. brucei*, but others appear to be absent (e.g. XPA and DDB2), perform functions outside of NER (e.g. XPC, XPD)^14^ or exist as paralogs (e.g. XPBz and XPB)^15^. Reverse genetics analyses have shown that TbCSB, TbXPBz, and TbXPG play roles in protection against UV-induced DNA damage and/or cisplatin DNA adducts, whereas TbXPB, TbXPD, and TbXPC do not^14,15^. Altogether, these findings suggest major divergences in NER relative to other eukaryotes, most notably, the apparent exclusion from NER of the multisubunit transcription factor TFIIH, whose core complex includes TbXPB and TbXPD but not XPBz^16,17^. Additionally, XPC and DDB factors are essential for cell viability and do not seem to act as DNA damage sensors, arguing against the existence of GG-NER in trypanosomes. Instead TC-NER would be the preferred and potentially the only NER pathway operating in *T. brucei*, likely due to the extent and predominance of RNA polymerase II polycistronic transcription in kinetoplastids^14^.

Here, we have characterized the *T. brucei* ortholog of XPF (TbXPF), an essential component of the NER pathway. The presence of the NER pathway in trypanosomes indicates that these parasites undergo replication and transcription-blocking DNA damage that must be repaired. Furthermore, XPF-deficient cells exhibit high sensitivity to various antitumor agents, providing proof of concept for the potential of NER inhibition as a strategy to enhance antiparasitic therapies.

## Results

### XPF from *Trypanosoma brucei*: role in the elimination of UV-induced DNA damage

Exposure of cells to UVC radiation leads to different types of DNA damage, primarily dipyrimidinic photolesions such as cyclobutane pyrimidine dimers (CPDs) and pyrimidine-(6,4)-pyrimidone photoproducts (6-4PPs), which are mainly repaired by the nucleotide excision repair (NER) pathway. While NER can be initiated either by global genome repair (GG-NER) or transcription-coupled repair (TC-NER), the final steps are common to both sub-pathways and involve, among other activities, incision at the 5′ side of the DNA damage by the XPF-ERCC1 nuclease. XPF and ERCC1 form a stable heterodimeric complex essential for NER. Using the homology detection tool BLAST and human proteins as query sequences, the *XPF* (*Tb927.5.3670*) and *ERCC1* (*Tb927.7.2060*) genes were identified in the *T. brucei brucei* TREU927 genome database (TriTrypDB).

**Fig. 1:**
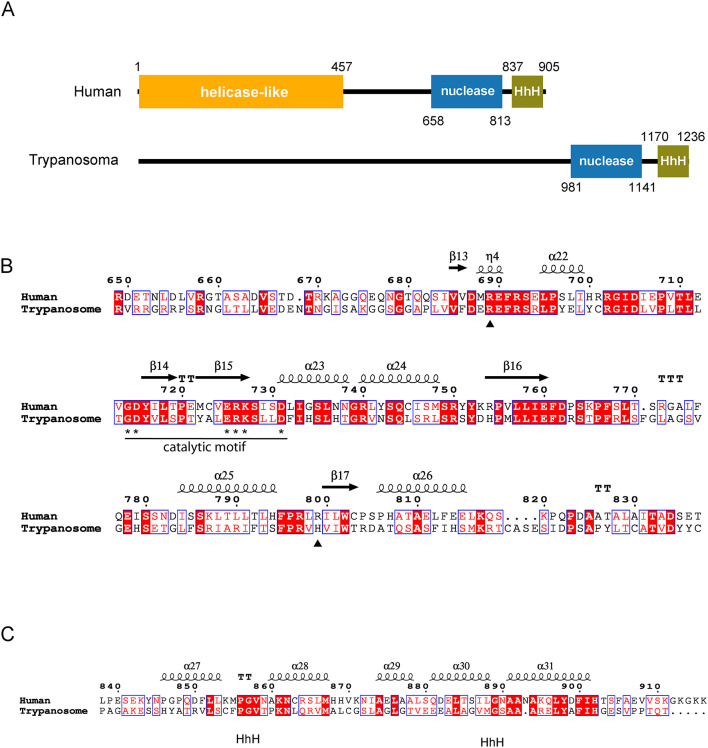
Comparative analysis of human and trypanosomal XPF proteins. (**A**) Schematic representation of XPF proteins. Human domains were represented according to UniProtKB/SwissProt (Q92889.3). Human XPF has a helicase-like domain that lacks key residues essential for ATP binding and hydrolysis activity. *T. brucei* protein domains were identified using the InterPro protein signature databases. No significant similarity to the human helicase-like domain was found in TbXPF (**B**) Sequence alignment of *T. brucei* and human XPF nuclease domains. The XPF nuclease domain extends from amino acid 658 to 813 of human XPF. Residues in this domain involved in catalysis (*) and those associated with clinical phenotypes (▲) are indicated. Mutation R689S was found in patient FA104 associated to Fanconi anemia disorder; patient XP42RO with Xeroderma pigmentosum was homozygous for mutation R799W^10^. (**C**) Sequence alignment of helix hairpin helix (HhH) domains. Sequence alignment was generated using MultAlin^57^ and the final format was obtained with ESPript^58^. XPF protein sequences were retrieved from NCBI RefSeq: *Homo sapiens* (NP_005227.1); *Trypanosoma brucei* (XP_845037.1).

To assess the functional significance of *T. brucei* XPF, we performed a detailed sequence alignment with the human ortholog, focusing on conserved structural and catalytic elements. Pairwise alignment using the EMBOSS Needle tool (EMBL’s European Bioinformatics Institute) revealed significant homology between trypanosomal and human XPF (*identity = 20.1%; similarity = 31.7%*). TbXPF is a protein of 1242 amino acids (136 kDa) with a well-conserved ERCC4 nuclease domain (Fig. 1A, B), including the metal-binding residues (*GDXn(V/I/L)ERKx3D* motif) that define the active members of this endonuclease family^18^. Two residues previously associated with clinical phenotypes in human XPF-related disorders are highlighted; one of these is conserved in *T. brucei*, supporting functional relevance of the active site, while the other is not conserved (Fig. 1B). In addition, we analyzed the tandem helix-hairpin-helix (HhH) domain, which in human XPF mediates dimerization with the equivalent domain in ERCC1 to form a functional endonuclease complex. This domain is also conserved in TbXPF (Fig. 1C), suggesting the potential for interaction with the ERCC1 homolog in *T. brucei*. These features support the notion that TbXPF retains key structural and functional characteristics of a canonical NER endonuclease.

**Fig. 2:**
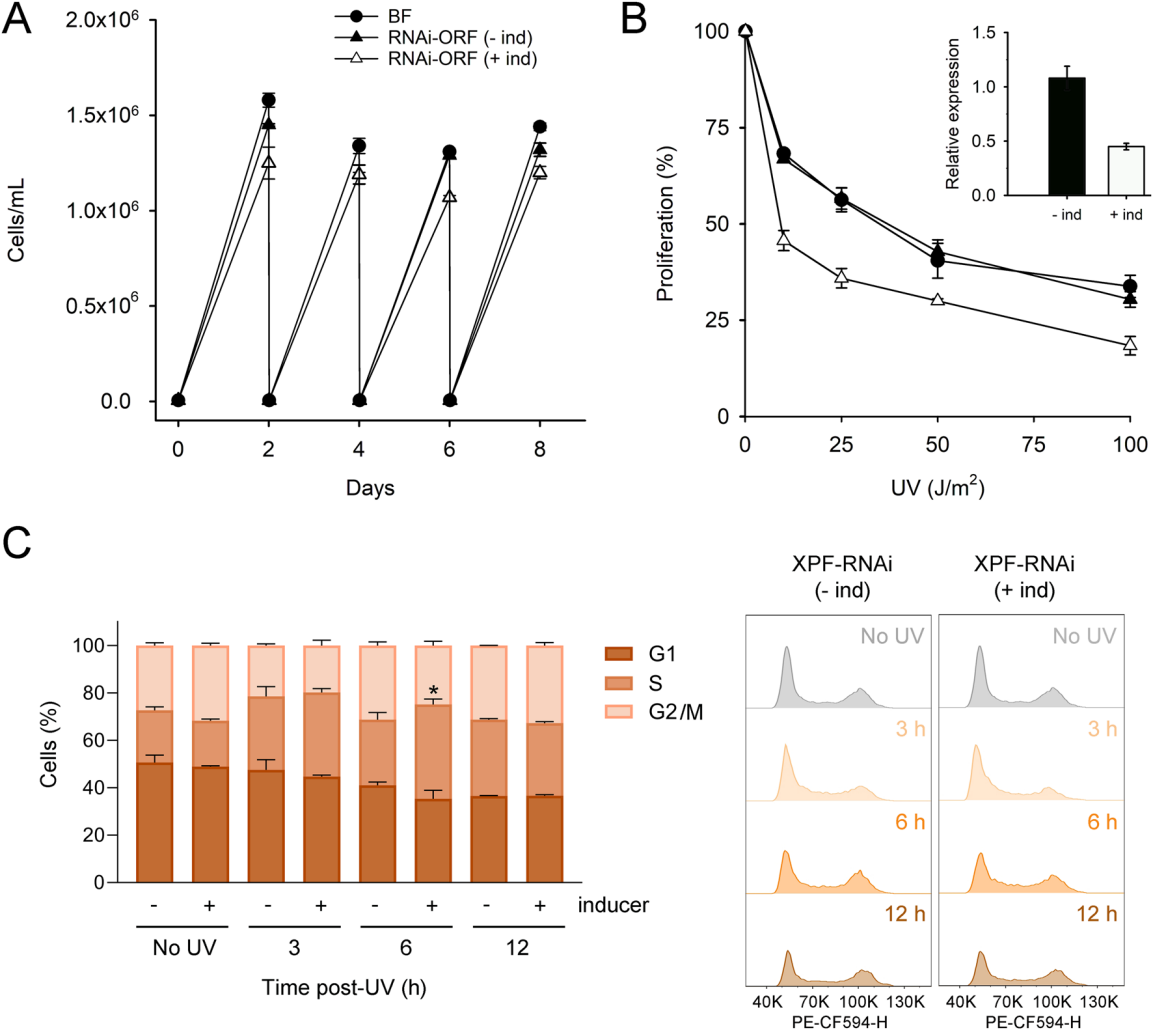
Impact of XPF on cell proliferation and cell cycle progression following UV irradiation. (**A**) Cell growth of trypanosomes cultured in standard HMI-9 medium over an eight-day period. RNAi-ORF: RNAi cell line targeting *XPF* open reading frame (**B**) UV sensitivity assay. Log-phase parasites were exposed to increasing doses of UVC irradiation and incubated for 48 h at 37 °C. Proliferation was calculated relative to cell growth of non-irradiated cells. RT-qPCR quantification of mRNA for the RNAi-targeted gene in the absence (-ind) or the presence of doxycycline (+ ind), relative to mRNA levels in parental bloodstream parasites. Represented data are the mean (± SD) of two independent determinations. (**C**) Effect of UVC irradiation and XPF depletion on cell cycle progression. Cells were exposed to UV light (50 J/m^2^) and samples collected at different times after irradiation. Cell cycle phase was determined by analyzing DNA content by flow cytometry (FACS). Plots show the percentage of cells in each of the different cell cycle stages: G1, S and G2/M. Right panel shows representative histograms used in the analysis. The proportion of cells with DNA content lower than G1 and higher than post-G2/M did not exceeded 10% in any experimental condition and the absence of XPF did not cause any significant difference. Represented data are the mean (± SD) of two independent determinations. Asterisk indicates statistically significant differences between induced and non-induced samples within the same experimental condition (*P < 0.05; unpaired t-test).

To investigate the role of the NER pathway in trypanosomes, an inducible stable RNAi cell line expressing a dsRNA against the ORF sequence of XPF was established (RNAi-ORF cell line). Upon induction, RNAi-ORF cells exhibited a 50% decrease in *XPF* mRNA levels without affecting normal proliferation. However, XPF-silenced cells were more sensitive to UV irradiation than non-induced and parental cells (Fig. 2A, B).

The impact of XPF on cell proliferation after UV exposure was assessed by monitoring cell cycle progression over 12 h (Fig. 2C). It is well established that bulky UV lesions, such as cyclobutane pyrimidine dimers (CPDs) and 6–4 photoproducts, can block the progression of replicative DNA polymerases during S phase, leading to replication fork uncoupling, stalling, and the activation of the DNA damage response^19^. Accordingly, an accumulation of cells in S-phase became evident 3 h post-irradiation in both repair-proficient and repair-deficient cells. By 6 h post-irradiation, while XPF-proficient cells had begun to recover, an accumulation in S-phase persisted in XPF-depleted parasites, pointing to impaired cell cycle progression.

**Fig. 3:**
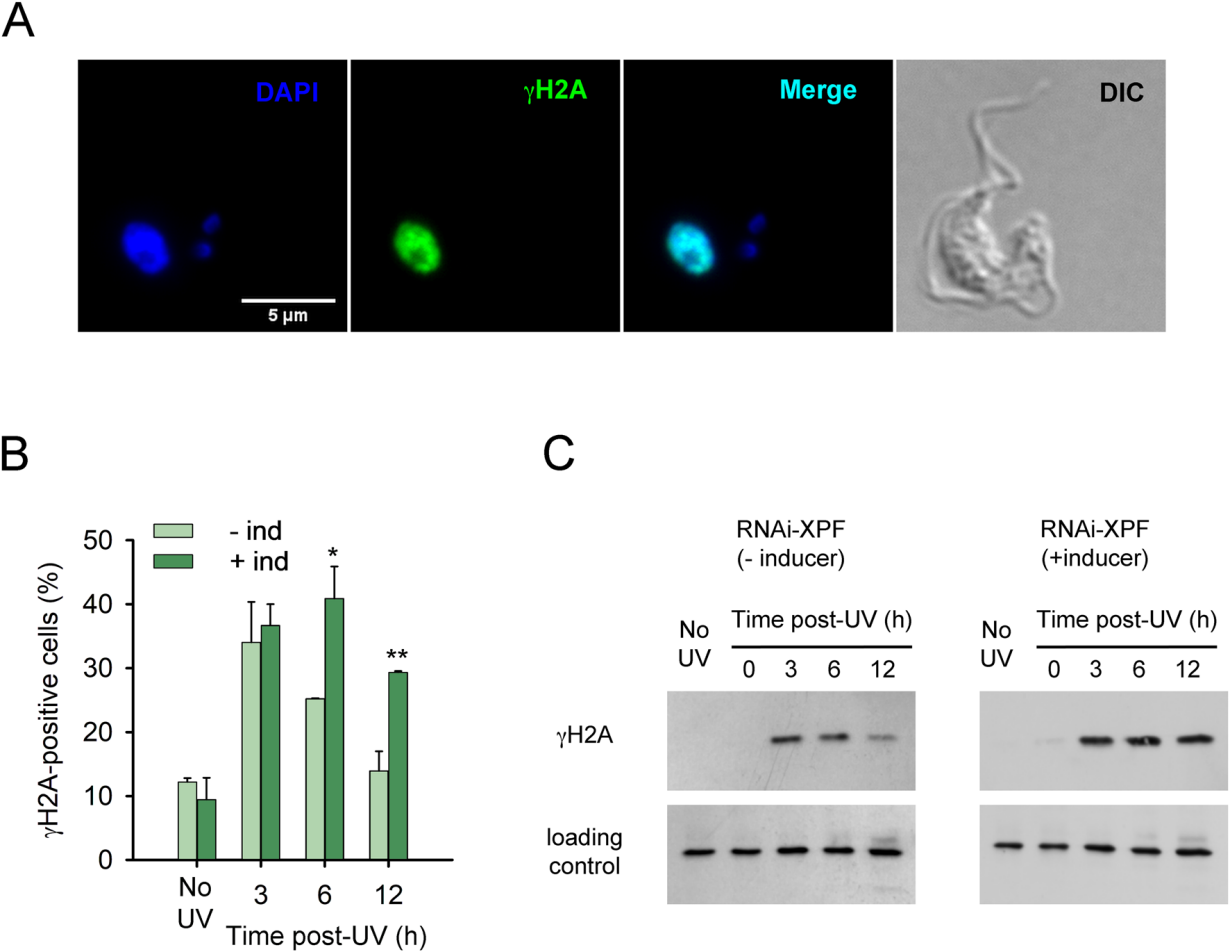
XPF depletion results in persistent γH2A signaling after UV-induced DNA damage (**A**) Representative immunofluorescence image of a γH2A-positive parasite at 6 h post-irradiation at 100x magnification. This example illustrates the characteristic pan-nuclear γH2A signal used to score positive cells in the quantification. Immunofluorescence analysis was carried out using an anti-γH2A primary antibody^56^ and an Alexa Fluor 488-conjugated anti-rabbit secondary antibody. Fluorescent images were captured using a Leica DMI8 inverted microscope. (**B**) Quantification of γH2A-positive cells over time after UV irradiation (25 J/m^2^), based on pan-nuclear γH2A staining by fluorescence microscopy (*n* > 100 per condition, two independent replicates). (**C**) Western blot showing γH2A levels. Whole cell extracts corresponding to approximately 5 × 10^6^ cells were collected at the indicated time points after UV irradiation (50 J/m^2^). Blots were probed with anti-γH2A antibody to detect histone H2A phosphorylation. Anti-*T. brucei* ITPase was used as a loading control^59^.

To assess the cellular response to UV-induced DNA damage, we examined the γ-phosphorylation of histone H2A (γH2A), a well-established marker of DNA damage signaling in trypanosomes^20^ (Fig. 3). While γH2A can be associated with DNA double-strand breaks, it also responds to other forms of genotoxic stress, including replication stress and the accumulation of single-stranded DNA regions, both of which are known consequences of UV irradiation^21^. Notably, the γH2A signal appeared predominantly as a diffuse, pan-nuclear staining pattern, consistent with previous observations describing UV-induced phosphorylation of H2A(X)^21^ (Fig. 3A). The percentage of γH2A-positive cells was quantified by fluorescence microscopy at various time points before and after UV irradiation to monitor the DNA damage response over time (Fig. 3B). Prior to irradiation, both control or XPF-deficient cells exhibited a low and comparable percentage of γH2A-positive nuclei. Following UV exposure, the number of γH2A-positive cells increased as early as 3 h in both induced and non-induced RNAi cells. Phosphorylation levels remained significantly higher in repair-deficient cells, even at 12 h post-irradiation, when the γH2A signal in control cells had already diminished to pre-irradiation levels. Western blot analysis further supported these observations, revealing elevated and sustained γH2A levels in XPF-depleted cells (Fig. 3C and S1). This increase likely reflects replication stress caused by unresolved UV-induced DNA lesions in the absence of XPF. These results underscore the importance of XPF in facilitating DNA damage resolution and maintaining genome integrity during recovery from UV-induced stress.

**Fig. 4:**
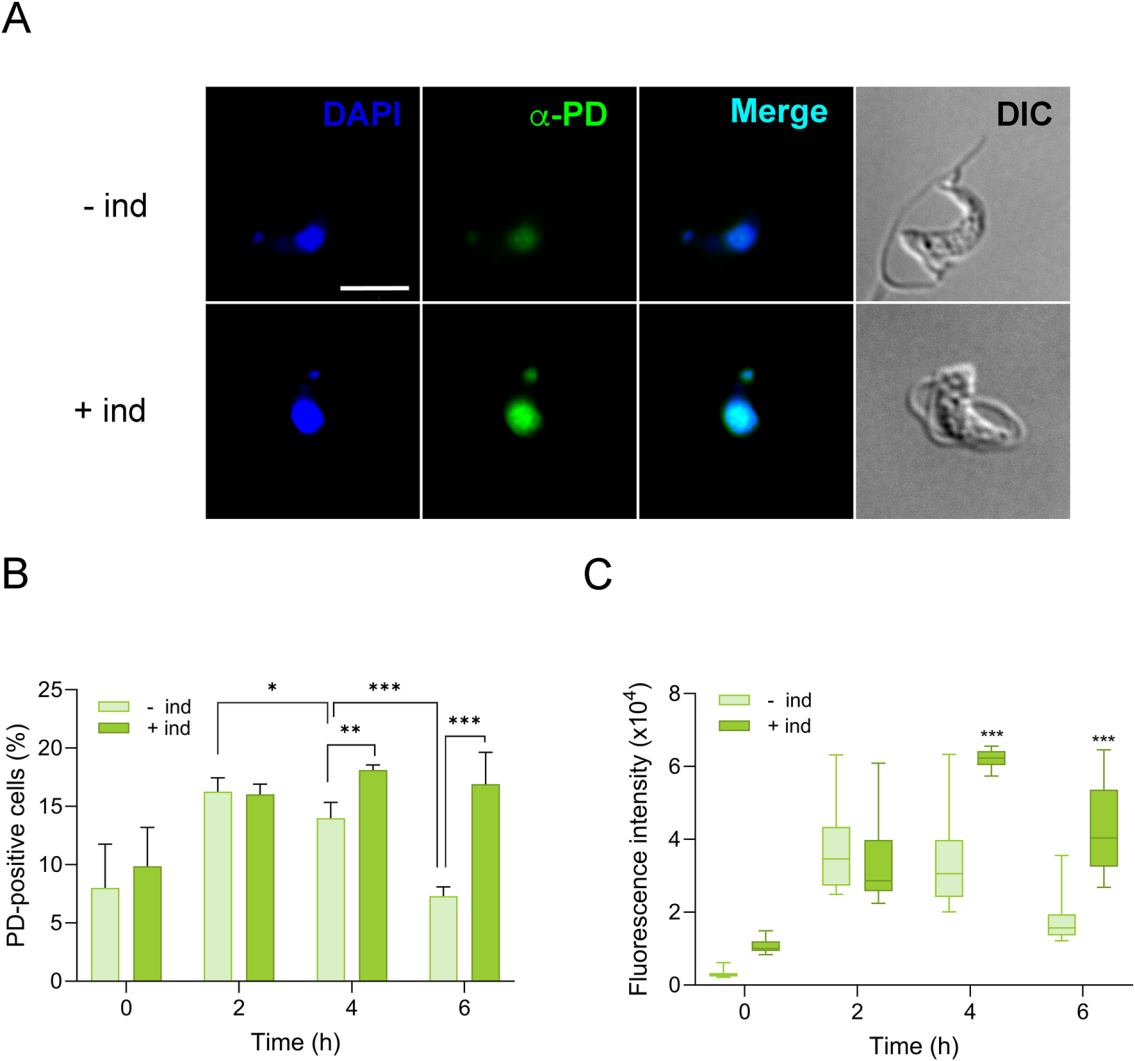
Repair of pyrimidine dimers is impaired in XPF-defective parasites. (**A**) Representative immunofluorescence images of *T. brucei* cells showing detection of pyrimidine dimers (PD) at 6 h post-irradiation with 50 J/m^2^ UVC. Images show a single cell from induced and non-induced XPF RNAi cultures. Individual channels for PD staining, DAPI, merge, and DIC are presented to illustrate the nuclear and kinetoplast localization of the PD signal and the difference in signal intensity between conditions. This example highlights the criteria used to define PD-positive cells. Scale bar corresponds to 5 µM. (**B**) Plot shows the percentage of PD-positive cells at each post-exposure sampling time. Microscopy images were captured and visually analyzed. A trained staff member identified and counted the cells until at least 1000 cells for each time point were registered. Represented data are the mean (± SD) of three independent determinations. (**C**) Fluorescence intensity associated to PD-positive cells. Represented data correspond to 50 cells showing maximal PD signal intensity for each experimental condition.

To determine whether *T. brucei* parasites are actually capable of repairing UV-induced DNA lesions, the removal of pyrimidine dimers (PDs) was assessed by immunofluorescence analysis using an anti-pyrimidine dimer antibody at different time points after UV irradiation (Fig. 4 and S2). An increase in PD levels was noted in both repair-proficient and -deficient parasites within the first few hours following UV irradiation. In control cells, PD staining peaked at 2 hours post-exposure to 50 J/m^2^ UVC, as shown by the proportion of PD-positive nuclei (Fig. 4A, B). While this may reflect delayed lesion formation, reminiscent of “dark CPDs” reported in mammalian cells due to melanin chemiexcitation^22 ^further studies are needed to determine whether a comparable process occurs in *T. brucei*. After the initial 2-hour peak, PD formation in control cells is likely offset by DNA repair, and the number of PD-positive nuclei decreased significantly at 4 and 6 h. In contrast, the proportion of PD-stained nuclei in XPF-deficient cells continued to rise beyond 2 hours and remained significantly elevated compared to control cells, consistent with a defect in lesion removal and impaired DNA repair capacity. A comparable trend was observed in fluorescence intensity between control and induced cells (Fig. 4A,C). Staining intensity was quantified as an indirect measure of UV photolesion burden per cell. While no significant differences in signal intensity were detected 2 hours after UV light exposure, the absence of a functional NER pathway correlated with higher levels of PD staining per nucleus at 4 and 6 h post-irradiation. Taken together, these findings provide strong molecular evidence for the involvement of the NER pathway in the removal of UV-induced DNA damage in trypanosomes.

### XPF repairs intra- and inter-strand DNA crosslinks

**Fig. 5:**
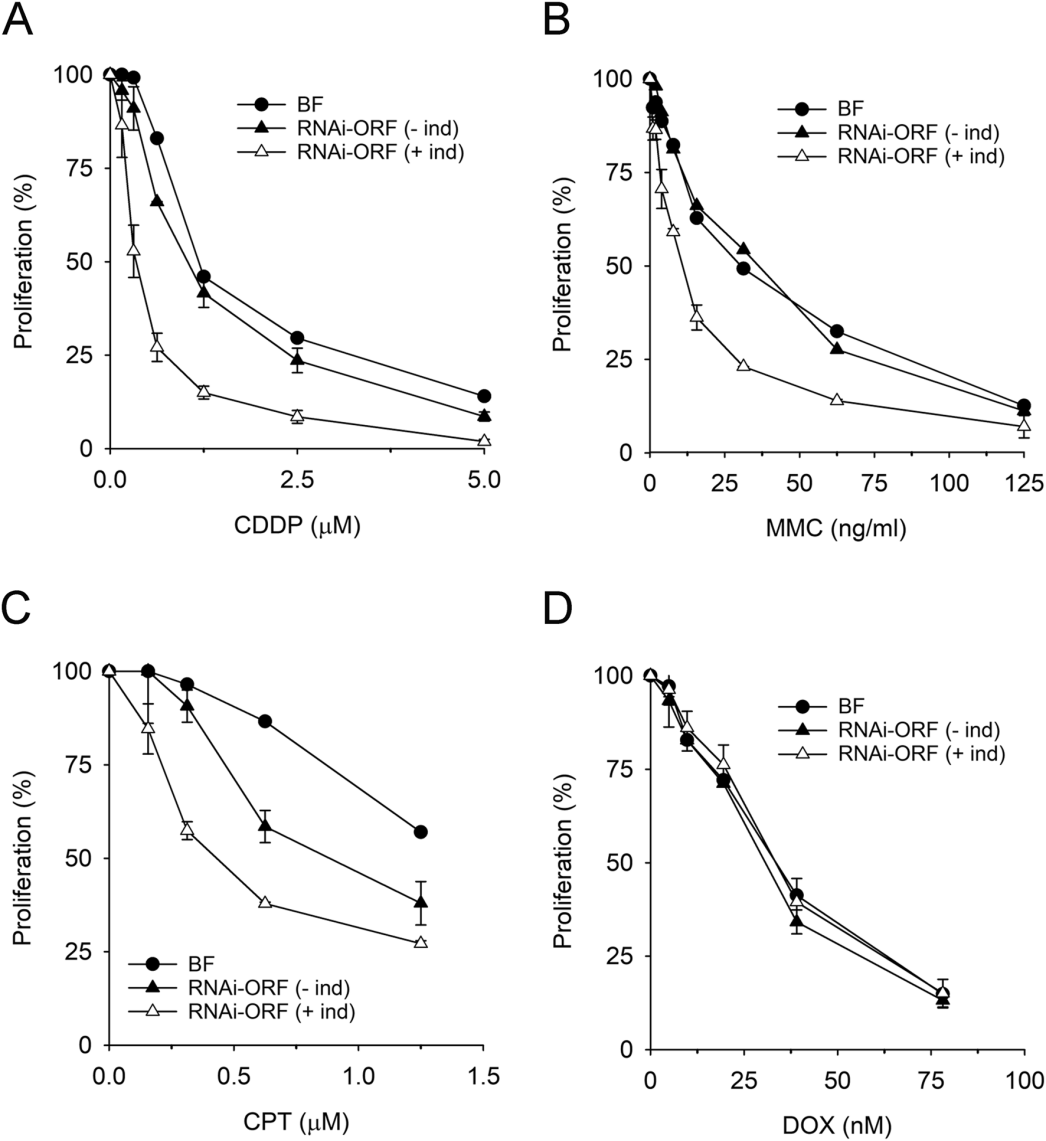
XPF-deficient cells exhibit increased sensitivity to anticancer agents. Log-phase parasites were exposed to increasing concentrations of (**A**) cisplatin (CDDP); (**B**) mitomycin C (MMC); (**C**) camptothecin (CPT); (**D**) doxorubicin (DOX) for 24 h at 37 °C. Cell growth was measured with resazurin as described in Methods. For each cell line, proliferation was calculated relative to cell growth in the absence of compound. Experiments were performed at least three times, in duplicate. Values are the mean (± SD).

In order to gain insight into the cellular function of TbXPF, RNAi-depleted cells were exposed to several DNA-damaging agents some of which are effective chemotherapy drugs used in the treatment various cancer types. Cisplatin (CDDP) is a front-line drug for lung, colorectal, ovarian, and head-and-neck cancers. In human cells, cisplatin-induced DNA lesions, mostly intra-strand diadducts^23^are primarily repaired by the NER pathway^24,25^. On the other hand, mitomycin C (MMC) is classified as an alkylating agent capable of covalently binding DNA and inducing inter-strand cross-linked lesions^26^. Inter-strand crosslinks (ICLs) exert their cytotoxic action by interfering with DNA replication and transcription^27^. As shown in Fig. 5A, B, both CDDP and MMC strongly impaired parasite proliferation, an effect that was even more pronounced in parasites with reduced levels of XPF. These results support a role for the trypanosomal XPF endonuclease in the repair of both intra- and inter-strand DNA crosslinks.

TbXPF was further investigated by exposing the cells to camptothecin (CPT) and doxorubicin (DOX), both considered topoisomerase poisons that ultimately generate DNA-protein crosslinks. CPT blocks the reaction catalyzed by DNA Topoisomerase I (Topo I), resulting in the accumulation of a covalent complex between the enzyme and the 3′ end of the cleaved DNA^28^. Conversely, DOX intercalates into unreplicated DNA and inhibits topoisomerase II (Topo II) activity, thereby blocking DNA unwinding during transcription^29^. Unlike Topo I-DNA crosslinks, Topo II-DNA covalent complexes involve phosphotyrosyl linkages with a 5’ DNA end. Both CPT and DOX are highly toxic to trypanosome cells (Fig. 5C, D). Down-regulation of XPF strongly increased sensitivity to CPT but not to DOX, suggesting that TbXPF participates in the processing of 3’-DNA-topoisomerase crosslinks or of those genotoxic intermediates that may result from them.

We also investigated the contribution of NER to fexinidazole (FEX) resistance. FEX, a 5-nitroimidazol derivative, is the first all-oral drug for *T. b. gambiense* sleeping sickness. It has been proposed that FEX nitroreduction results in the formation of reactive chemical species with the potential to react with DNA and cause genetic damage^30^. As shown in Fig. S3A, XPF does not confer resistance to FEX, ruling out the generation of NER substrates by this drug as part of its mechanism of action. Other types of DNA damage such as DNA single-strand breaks and oxidative base damage, were also not XPF repair substrates, as RNAi-silenced parasites behaved identical to parental cells when exposed to phleomycin (PLM) or hydrogen peroxide (H_2_O_2_) (Fig. S3B, C).

### Subcellular localization of TbXPF

**Fig. 6:**
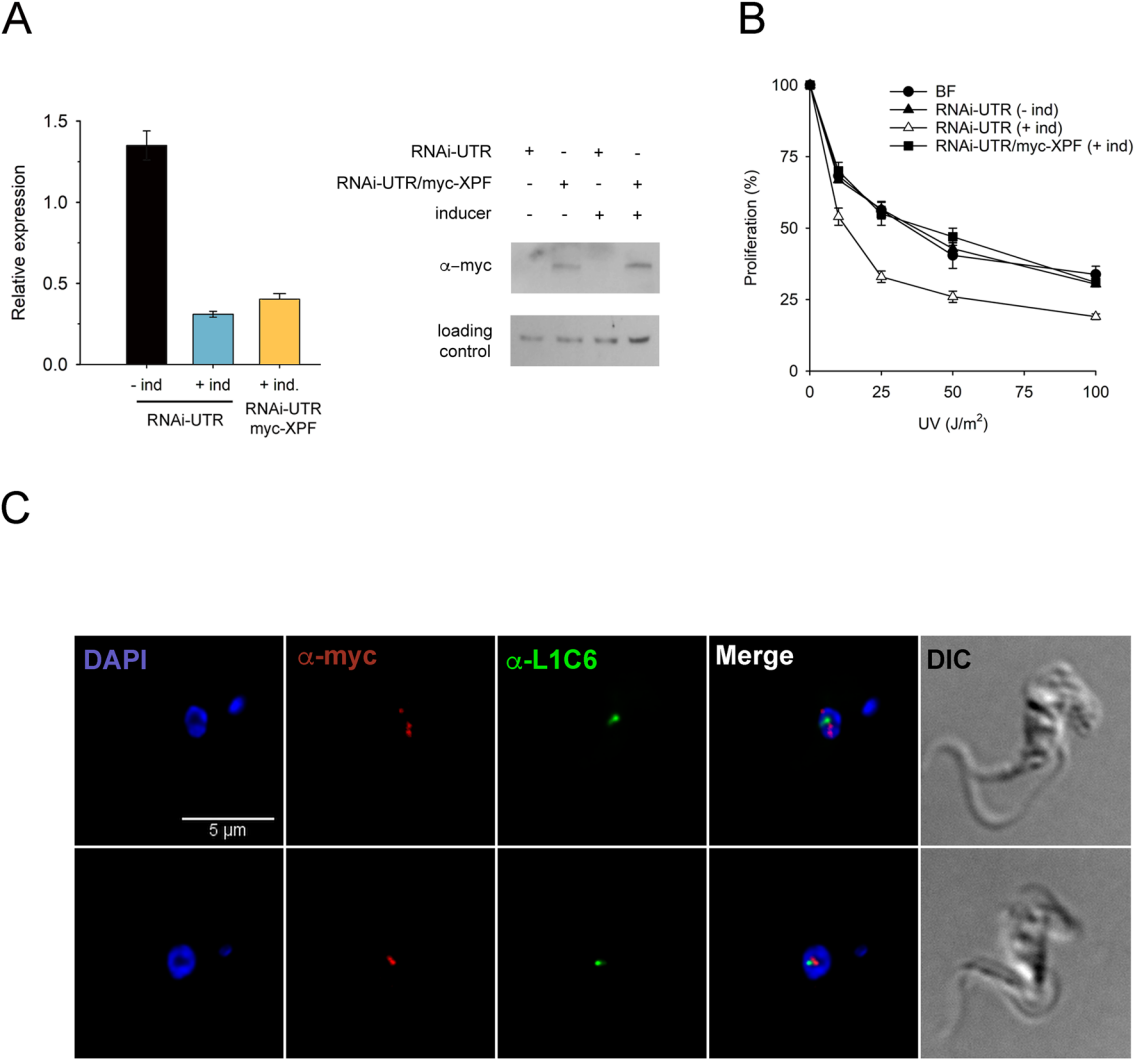
XPF localizes to the nucleus in bloodstream *T. brucei*. (**A**) Left, levels of mRNA were determined by RT-qPCR for each cell line and calculated relative to mRNA levels in parental bloodstream form; Represented data are the mean (± SD) of two independent determinations. Right, western blot showing myc-TbXPF protein levels in whole cell extracts from 5 × 10^6^ parasites. Myc-TbXPF was detected with anti-myc monoclonal antibodies. As loading control, an anti-TBAPE1 polyclonal antibody was used^37^. Images were cropped and aligned for easier comparison. Samples and controls derived from the same experiment. Blots were generated through sequential incubations with the different antibodies. Original blots are shown in Fig. S4 (**B**) Log-phase parasites were exposed to UVC irradiation and incubated for 48 h at 37 °C in HMI-9 medium before counting. RNAi and protein expression were pre-induced by adding doxycycline (1 µg/mL) to the corresponding cell lines 48 h before the experiment. Proliferation was calculated relative to cell growth in non-irradiated cells. Experiments were performed three times, values are the mean (± SD). RNAi-UTR: RNAi cell line targeting *XPF* 3’-UTR; RNAi-UTR/myc-XPF: transgenic cell line that expresses an inducible myc-tagged version of TbXPF in cells where the RNAi targets the 3’-UTR of *XPF.* (**C**) Immunofluorescence microscopy images were obtained to determine the subcellular localization of myc-TbXPF. Nuclear and kinetoplast DNA were stained with DAPI. Myc-TbXPF was visualized with anti-myc tag rabbit monoclonal antibody (clone 71D10) and Alexa Fluor 594 goat anti-rabbit. Nucleolar protein L1C6 was detected with anti-L1C6 mouse monoclonal antibody and Alexa Fluor 488 goat anti-mouse. Images were collected with an inverted Leica DMi8 microscope, 100x objective, and LASX software.

The subcellular distribution of the trypanosomal XPF ortholog was determined using a transgenic cell line that expresses an inducible myc-tagged version of TbXPF in cells where the RNAi targets the 3’-UTR of XPF (RNAi-UTR/myc-XPF cell line). Upon induction, endogenous *XPF* mRNAs are depleted, while ectopic myc-TbXPF, which lacks the UTR sequences, escapes silencing (Fig. 6A). As expected, RNAi-UTR induced cells exhibit an UV-sensitive phenotype, while the expression of myc-TbXPF restores normal proliferation (Fig. 6B). The phenotypic rescue by myc-TbXPF validates the myc-tagged version as a functional protein suitable for subcellular localization studies. Indeed, staining with anti-myc antibodies showed that XPF is present in the nuclear compartment (Fig. 6C), a localization that is consistent with its DNA repair function. A closer inspection revealed XPF expression in several nucleoplasmic and nucleolar foci (Fig. 6C, upper row). In those cases where only one focus was detected in the nucleus, it was present in the nucleolus (Fig. 6C, lower row). A similar nuclear distribution was observed using a different primary anti-myc antibody (Fig. S5). Nucleolar localization was confirmed by double-staining of XPF and the nucleolar protein L1C6^31^, which showed both proteins in close proximity (Fig. 6C). In mammalian cells, various DNA repair proteins have been reported to associate with the nucleolus. However, it remains unclear whether the nucleolus merely acts as a storage site for DNA damage response proteins or if these proteins perform specific nucleolar roles^32^,

### N3-Alkylation DNA damage in trypanosomes is repaired by NER

Monofunctional SN2-alkylating agents such as methyl methanesulfonate (MMS), primarily induce N-alkylation of purine bases in DNA. N3-methyladenine (N3-meA) adducts are the main cytotoxic lesion induced by MMS, and if left unrepaired, they can block DNA replication, cause further DNA damage and ultimately lead to cell death^33^. N3-meA is typically repaired by the base excision repair (BER) pathway, which is initiated by a methyladenine DNA glycosylase that excises the damaged base, creating an abasic site^1^. Accordingly, prokaryotic and eukaryotic cells deficient in apurinic/apyrimidinic (AP) endonuclease activity exhibit increased sensitivity to the toxic effects of MMS due to their inability to repair alkylation damage via BER^34,35^. On the other hand, the O6-position of guanine is a major site of methylation by SN1-type alkylating agents such as methyl-nitro-nitrosoguanidine (MNNG). While O6-methylguanine (O6-meG) lesions occur at a lower frequency than N-methyl adducts, they are responsible for many of the cytotoxic biological effects of alkylating agents. O6-meG adducts can be directly reversed to guanine by the action of a methylguanine methyltransferase protein^36^.

**Fig. 7:**
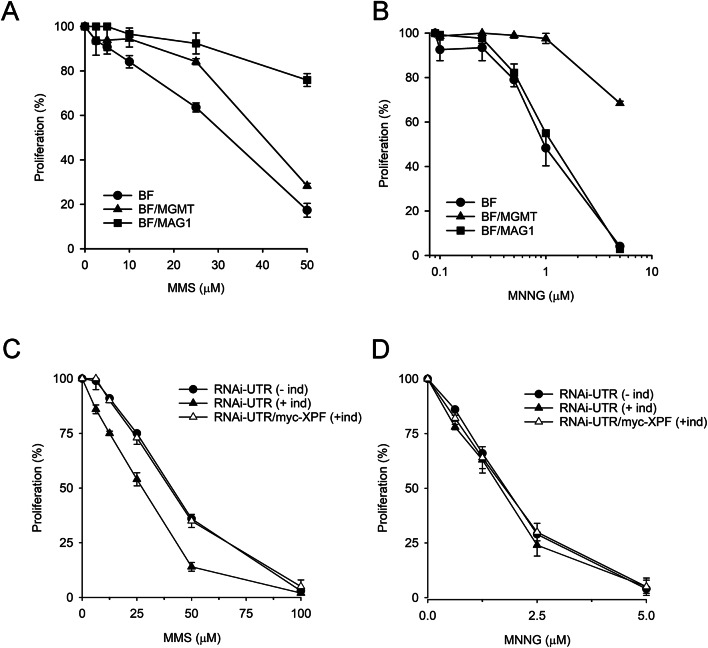
Trypanosomal NER is involved in the repair of N3-methylA adducts. (**A**,**B**) Assessment of DNA damage induced by SN1 and SN2 methylating agents. Parental (BF) and transgenic bloodstream *T. brucei* cell lines expressing MAG1 (BF/MAG1) or MGMT (BF/MGMT) were exposed to increasing concentrations of methyl methanesulfonate (MMS) or methyl-nitro-nitrosoguanidine (MNNG) for 48 h at 37 ºC. (**C**,**D**) Sensitivity of XPF-deficient parasites to MMS and MNNG. Proliferation was calculated relative to cell growth in the absence of compound. Experiments were performed at least three times, in duplicate. Values are the mean (± SD).

To determine the cytotoxic impact of the two main genotoxic lesions induced by methylating agents –N3-meA and O6-meG– on *T. brucei*, we analyzed the parasite’s sensitivity to MMS and MNNG. The specific impact of each DNA lesion can be inferred from the response of cells expressing *Saccharomyces cerevisiae* methyladenine glycosylase 1 (MAG1) or human methylguanine methyltransferase (MGMT) to these methylating agents (Fig. S6). MAG1 recognizes and repairs N3-meA, while MGMT specifically repairs O6-meG lesions. As shown in Fig. 7A, the impaired cell proliferation induced by MMS was rescued by the overexpression of MAG1 and, to a lesser extent, MGMT, indicating that both N3-meA and O6-meG contribute to the cytotoxic effect. In contrast, only MGMT was able to revert the sensitivity to MNNG, confirming that its cytotoxicity is exclusively due to O6-meG adduct formation (Fig. 7B).

We previously reported that *T. brucei* parasites deficient in AP endonuclease activity do not exhibit an increased sensitivity to the toxic effects of MMS^37^. To investigate whether trypanosomes use NER instead of BER for the removal of methyl DNA adducts, XPF-deficient cells were treated with MMS and MNNG. Cell proliferation curves show that XPF depletion increases sensitivity to MMS, indicating that N3-methylA DNA lesions are indeed repaired by the NER pathway (Fig. 7C). Conversely, XPF does not confer resistance to MNNG, suggesting that O6-meG lesions are not physiological substrates for trypanosomal NER (Fig. 7D).

## Discussion

Nucleotide excision repair is the main pathway used by prokaryotic and eukaryotic organisms to remove a wide range of bulky DNA lesions such as those formed by UV light. In essence, most of the eukaryotic NER genes are present in the genome of kinetoplastid parasites, suggesting the conservation of practically all NER functions, including the strategies for DNA damage detection that define global-genome and transcription-coupled repair sub-pathways. However, early studies showed a specialization of these parasites at transcription-coupled repair, highlighting the functional divergence of many trypanosomal NER factors^14,15^.

Among the most striking differences is the apparent absence of the key NER scaffold protein XPA, raising important questions about how this pathway is organized in trypanosomes. XPA is a central factor in the coordination of NER through the interaction with almost all NER proteins including TFIIH, RPA, PCNA, XPC, DDB2 and ERCC1-XPF^3^. Human ERCC1 interacts with XPA through its central domain and facilitates the recruitment of the endonuclease to the 5′ junction at photoproducts during NER^38,39^. The absence of XPA in trypanosomes raises the question as to how NER is coordinated in this parasites and specifically how the complex XPF-ERCC1 is targeted to the DNA damage site. It is plausible that a protein structurally unrelated to XPA, yet exhibiting analogous properties, could take on the role of XPA during NER^40^.

The heterodimer formed by XPF and ERCC1 is a highly conserved protein complex, which has been identified and characterized in different model organisms. For instance, *S. cerevisiae* Rad1-Rad10 null mutants are viable^41^ and the activity of XPF-ERCC1 does not seem to be essential either in plants, worms or flies where viable *RAD1* mutants have been obtained and characterized^42–44^. In contrast, the animal models exhibit far more severe phenotypes. ERCC1- and XPF-deficient mice are not viable or undergo early postnatal death depending on the genetic background while in humans, mutations affecting these genes have been linked to some severe diseases, including the skin cancer-prone disease xeroderma pigmentosum (XP), a progeroid syndrome of accelerated aging (XFE), and cerebro-oculo-facio-skeletal syndrome (COFS)^45^. Similar to other lower eukaryotes, strong down-regulation of *XPF* mRNA levels did not have an apparent impact on *T. brucei* viability or cell proliferation. This observation is in agreement with a high-throughput phenotypic screen study using RNAi target sequencing (RIT-seq) which reported that XPF depletion does not impair significantly the growth of bloodstream or procyclic trypanosomes^46^. A similar lack of growth defect was also observed by Machado *et al.*^14^who showed that RNAi against various NER factors, including CSB and XPBz, did not significantly affect proliferation but did sensitize cells to genotoxic agents. Notably, XPG, like XPF, is one of the two NER nucleases. While its depletion did not impact cell growth, it conferred sensitivity specifically to cisplatin, suggesting a possible role in the transcription-coupled NER (TC-NER) subpathway. These findings, together with our results, support the notion that both XPG and XPF function within TC-NER. In our study, depletion of XPF had no major effect on proliferation but led to strong sensitivity to UV irradiation and slower repair kinetics of UV photolesions, highlighting its specific role under genotoxic stress. These observations are also consistent with previous studies in yeast and mice^45,47^ and further validate the requirement of the XPF-ERCC1 complex in the NER pathway of trypanosomes.

Beyond UV-induced DNA damage, TbXPF confers protection from inter-strand DNA crosslinks and TopoI-DNA complexes, indicating that besides NER, it plays a role in other DNA repair processes. Both human XPF-ERCC1 and yeast Rad1-Rad10 have been proposed to incise 5′ to the ICL lesion and initiate the unhooking step of ICL repair. Additionally, they are involved in repairing Topo I-induced DNA damage as an alternative pathway to the single-strand break repair enzyme tyrosyl-DNA phosphodiesterase 1 (Tdp1)^48,49^. Tdp1 processes Topo I-DNA adducts by cleaving the phosphodiester bond between the tyrosine of topoisomerase and the 3′-phosphate of DNA^50^. In yeast, inactivation of Tdp1 has little impact on CPT cytotoxic action except in the Rad1-Rad10 null genetic background^49^. Kinetoplastid genomes code for Tdp1 repair enzymes, but few studies are available on them, except for the Tdp1 enzyme in the parasite *Leishmania donovani*^51^. Further investigation is required to determine whether XPF-ERCC1 and Tdp1 functions are also redundant in trypanosomes.

Consistent with previous studies^14,15 ^this study provides multiple lines of evidence that demonstrate the presence of an active NER pathway in *T. brucei.* First, RNAi-mediated silencing of the *T. brucei XPF* gene, which codes for an essential component of the NER pathway, sensitizes cells to UV irradiation and other genotoxic agents that induce potential NER substrates. We show that UV-induced DNA lesions are actually being eliminated from DNA and not merely tolerated by translesion DNA synthesis. Moreover, XPF-defective trypanosomes present slower repair kinetics than repair-proficient cells, confirming the role of NER in lesion removal. The presence of the NER pathway in trypanosomes suggests that this parasite is susceptible to undergo replication and transcription-blocking DNA damage *in vivo*. Further investigation will help elucidate the exact nature of the physiological NER substrates and determine whether XPF function is essential in the mammalian host, where the accumulation of unrepaired DNA damage may challenge parasite survival.

Under genotoxic stress, genomic stability and protozoan survival may greatly depend on DNA repair systems such as NER, making them potential targets for chemotherapeutic interventions. Chemical inhibitors of NER, such as small molecules targeting XPF-ERCC1 interactions or endonuclease activity, could sensitize parasites to DNA-damaging agents. Previous research in other eukaryotic systems has identified potential NER inhibitors^52 ^and assessing their efficacy against *T. brucei* could open new possibilities for drug development. Future work should focus on identifying druggable vulnerabilities within the NER pathway that could be exploited to enhance the effectiveness of current antiparasitic treatments.

## Methods

### Growth and generation of *T. brucei* cell lines

All cell lines used in this work derive from the *Trypanosoma brucei brucei* single marker bloodstream form (BF) provided by George Cross’s laboratory^53^. Bloodstream cells were cultured at 37 ºC and 5% CO_2_ in HMI-9 medium supplemented with 10% (v/v) of fetal bovine serum (FBS) and 2.5 µg/mL Geneticin (Gibco). TbXPF coding sequence was amplified by PCR using *T*. *brucei* 427 genomic DNA as template and primers with sequences obtained from TriTrypDB (Tb427_050043200, ERCC4 domain containing protein). MAG1 open reading frame was amplified from pGAD424-MAG1, a gift from Dr. McIntyre (Institute of Biochemistry and Biophysics, Polish Academy of Sciences, Poland) while MGMT was amplified from human cDNA. TbXPF, MAG1 and MGMT were cloned into pGRV23-myc expression vector^37^. To inhibit XPF expression by RNA interference, two genetic constructs targeting either the coding (ORF) or the 3’-UTR regions of XPF were generated. In these plasmids the target DNA sequences of 500–600 base pairs approximately, were cloned in sense and antisense directions flanking a stuffer fragment. The stem-loop cassette was produced and integrated into pGR19 vector^54^ previously digested with HindIII and HpaI enzymes using an In-Fusion HD Cloning Kit (Takara Bio) following the manufacturer’s instructions. All primers used in this work are listed in Supplementary Table 1.

Ten micrograms of linear targeting DNA fragments obtained by NotI digestion were transfected into bloodstream parasites by electroporation using the Amaxa™ Human T Cell Nucleofector Kit (Lonza) and the Amaxa Nucleofector device. Clones were selected with the appropriate selection drugs at the following concentrations: 0.1 µg/mL puromycin and 5 µg/mL hygromycin (Sigma-Aldrich). RNAi and TbXPF expression was induced using 1 µg/mL of doxycycline (Sigma-Aldrich). Selected clones were analyzed by RT-qPCR or Western blot analysis.

### Sensitivity assays

To determine the sensitivity of *T. brucei* cells lines to different genotoxic agents, a Resazurin Reduction Cell Viability Assay was performed in 96-well plates. Mid-log phase *T. brucei* parasites were diluted to a working cell density so that the final cell concentration was 75,000 cells/mL, and 90 µL/well was dispensed into 96-well flat-bottom transparent assay plates. Compounds at different concentrations were added to the cell plates (10 µL/well) which were incubated for 24 h at 37 °C and 5% CO_2_. Four hours prior to the end of the incubation, 20 µL of a Resazurin solution (440 µM, Sigma-Aldrich) in pre-warmed HMI-9 was added to each well. Fluorescence was then measured in an Infinite F200 plate reader (TECAN infinite 200) at 550 nm (excitation filter) and 590 nm (emission filter). Assays were performed in duplicate at least three times to achieve a minimal *n* = 3 per dose-response. Camptothecin, phleomycin, mitomycin C, methyl methanesulfonate, hydrogen peroxide, cisplatin, doxorubicin (Sigma-Aldrich), methyl-nitro-nitrosoguanidine (MedchemExpress) and fexinidazole (Selleck) were used in this study. For UV irradiation, log-phase parasites at 5 × 10^3^ cells/mL in HMI-9 with 10% FBS were seeded in 60 mm Petri dishes prior to UVC irradiation (254 nm) at the indicated dose (UVP CL-1000 UV Crosslinker). After irradiation, plates were further incubated for 48 h at 37 ºC before cells were counted using a Z1 Coulter counter.

### RNA extraction and real time quantitative PCR (RT-qPCR)

Total RNA was extracted from 5 × 10^7^ parasites using the NucleoSpin RNA kit (Macherey-Nagel) according to the manufacturer’s instructions. Reverse transcription was performed using iScript™ cDNA Synthesis kit (Bio-Rad) and quantitative PCR assays were carried out in a iCycler IQ real-time PCR detection system (Bio-Rad), with 1X SYBR Green master mix (Thermo Scientific). Specific primers were designed to amplify *TbXPF* (Table S1) and their efficiency was calculated from the equation E = 10^[− 1/slope]^ obtained after plotting the cycle threshold (Ct) values *versus* the log of the cDNA amount^55^. Relative expression of each gene was calculated with respect to the expression of a reference gene (*actin A*, Tb927.9.8850). Two independent experiments were performed and sample triplicates were used in all RT-qPCR assays.

### Immunofluorescence analysis

Intracellular localization of TbXPF was studied by immunofluorescence microscopy. A total of 5 × 10^3^ cells were spread onto poly-L-lysine-coated slides. The cells were then fixed with 4% paraformaldehyde (diluted in 1X PBS) for 20 min at room temperature, washed twice, and permeabilized with 1% IGEPAL (Sigma-Aldrich) for 40 min. Next, cells were blocked with 5% blocking reagent (Roche) for 30 min. The coverslips were first incubated with the primary antibody in blocking solution (1X PBS, 0.5% blocking reagent) for 1 h and washed three times for 10 min. The primary antibodies used in this study were: mouse monoclonal anti-myc tag (clone 4A6) (1:200 dilution; Sigma-Aldrich); rabbit monoclonal anti-myc tag antibody (clone 71D10) (1:200 dilution; Cell Signalling Technology); mouse monoclonal anti-L1C6 (1:200 dilution; a gift from Dr. Bastin and Dr. Glover, Institut Pasteur, France). H2A phosphorylation was detected using an anti-γH2A primary antibody^56^. Following incubation with the primary antibody, cells were incubated with Alexa Fluor 488 goat anti-mouse IgG or Alexa Fluor 594 goat anti-rabbit IgG secondary antibodies (Sigma-Aldrich, 1:1000 diluted in blocking solution) for 1 h. After washing, coverslips were dehydrated in methanol for 1 min and stained and mounted with Vectashield-DAPI (Vector Laboratories, Inc.). For intracellular localization studies, vertical stacks of up to 40 slices (0.2 μm steps) were captured using an inverted Leica DMi8 microscope, 100X objective, and LASX software. Images were deconvolved and pseudo-colored with Huygens Essential software (version 3.3; Scientific Volume Imaging) and processed with ImageJ software (version 1.37; National Institutes of Health).

### DNA damage detection by Immunofluorescence microscopy

To analyze DNA damage content by immunofluorescence microscopy, parasites were fixed in 4% paraformaldehyde (diluted in 1X PBS) for 20 min at room temperature, washed twice and permeabilized with 1% IGEPAL (Sigma-Aldrich) for 40 min. For pyrimidine dimer detection, tyramide-based signal amplification was performed using the Alexa Fluor™ 488 Tyramide SuperBoost™ (goat Anti-Mouse IgG, Invitrogen), following the manufacturer’s instructions. Briefly, endogenous peroxidases were quenched using a 3% hydrogen peroxide solution for 1 h to reduce non-specific tyramide activation. DNA was then denatured with 2 M HCl for 30 min at room temperature. Samples were then washed five times, and blocking was performed using the blocking solution included in the kit. To detect UV-induced DNA lesions cells were incubated with anti-thymine dimer antibody (mouse monoclonal; Sigma-Aldrich) followed by a secondary fluorescent antibody (Poly-HRP-conjugated Alexa Fluor 488 Goat Anti-Mouse IgG, Invitrogen). Cells were then incubated with the tyramide working solution for 10 min at room temperature to enable tyramide signal amplification. Samples were mounted using Vectashield-DAPI mounting medium (Vector Laboratories, Inc.). Fluorescent images were captured using a Leica DMI8 inverted microscope with a 40 × 1.3 NA objective. At least 500 cells per experimental condition were analyzed, and the percentage of DNA damage positive nuclei was determined visually. To quantify the green fluorescence signal, the mean grey value was measured for each cell using an automated macro that segmented the cells of interest.

### Fluorescence-activated cell sorting (FACS) analysis

Parasites were harvested by centrifugation (1000 × *g*, 4 °C, 10 min) in mid log-phase (1 × 10^6^ cells/mL). Samples were washed twice in 1X PBS, resuspended in 70% ice-cold ethanol/PBS solution and fixed overnight at 4 ºC. Cells were stained and treated with 40 µg/mL of propidium iodide and 10 µg/mL of RNase A, respectively, in 500 µl of 1X PBS during 30 min at room temperature in the dark. FACS analysis of the cell cycle was carried out with a FACSCalibur flow cytometer (Becton Dickinson) and FlowJo software v10.0.

### Statistics

All the data analyses were performed with GraphPad Prism 8 (GraphPad Software) and SigmaPlot Software 15.0 (Grafiti LLC) and results were expressed as mean ± SD of at least three experiments unless indicated. Comparisons between two groups were analyzed by unpaired two-tailed t-tests. A value of *P* < 0.05 was considered statistically significant. * *P* < 0.05, ** *P* < 0.01, *** *P* < 0.001.

## Electronic supplementary material

Below is the link to the electronic supplementary material.

Supplementary Material 1

## Data Availability

The authors declare that the data supporting the findings of this study are available within the article and its supplementary information files.
