# Supplementary material for: Role of the nucleotide excision repair endonuclease XPF in the kinetoplastid parasite Trypanosoma brucei
Source: Sci Rep. 2025 Jul 2;15:23579. doi: 10.1038/s41598-025-08659-y (PMC12222486; doi:10.1038/s41598-025-08659-y)
Supplement: Supplementary file 1 — Supplementary Material 1 [file 41598_2025_8659_MOESM1_ESM.pdf]

SUPPLEMENTARY INFORMATION

Role of the Nucleotide Excision Repair Endonuclease XPF in the Kinetoplastid Parasite *Trypanosoma brucei*

Claudia Gómez-Liñán, María Sáez-Maldonado, Laura Montosa-Hidalgo, Luis Miguel Ruiz-Pérez, Dolores González-Pacanowska and Antonio E. Vidal

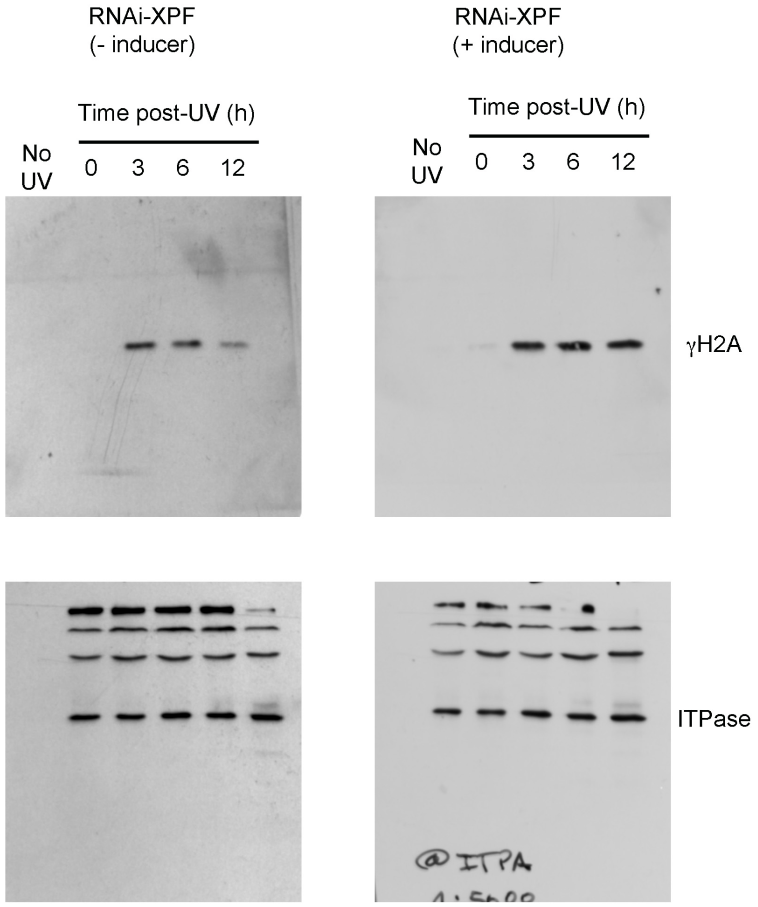

Figure S1. Full size images of western blots presented in Figure 3 (panel C) of the manuscript. Western blot showing γH2A levels. Whole cell extracts corresponding to approximately 5 x 10^6 cells were collected at the indicated time points after UV irradiation (50 J/m²). Blots were probed with anti-γH2A antibody to detect histone H2A phosphorylation. Anti-*T. brucei* ITPase was used as a loading control [63].

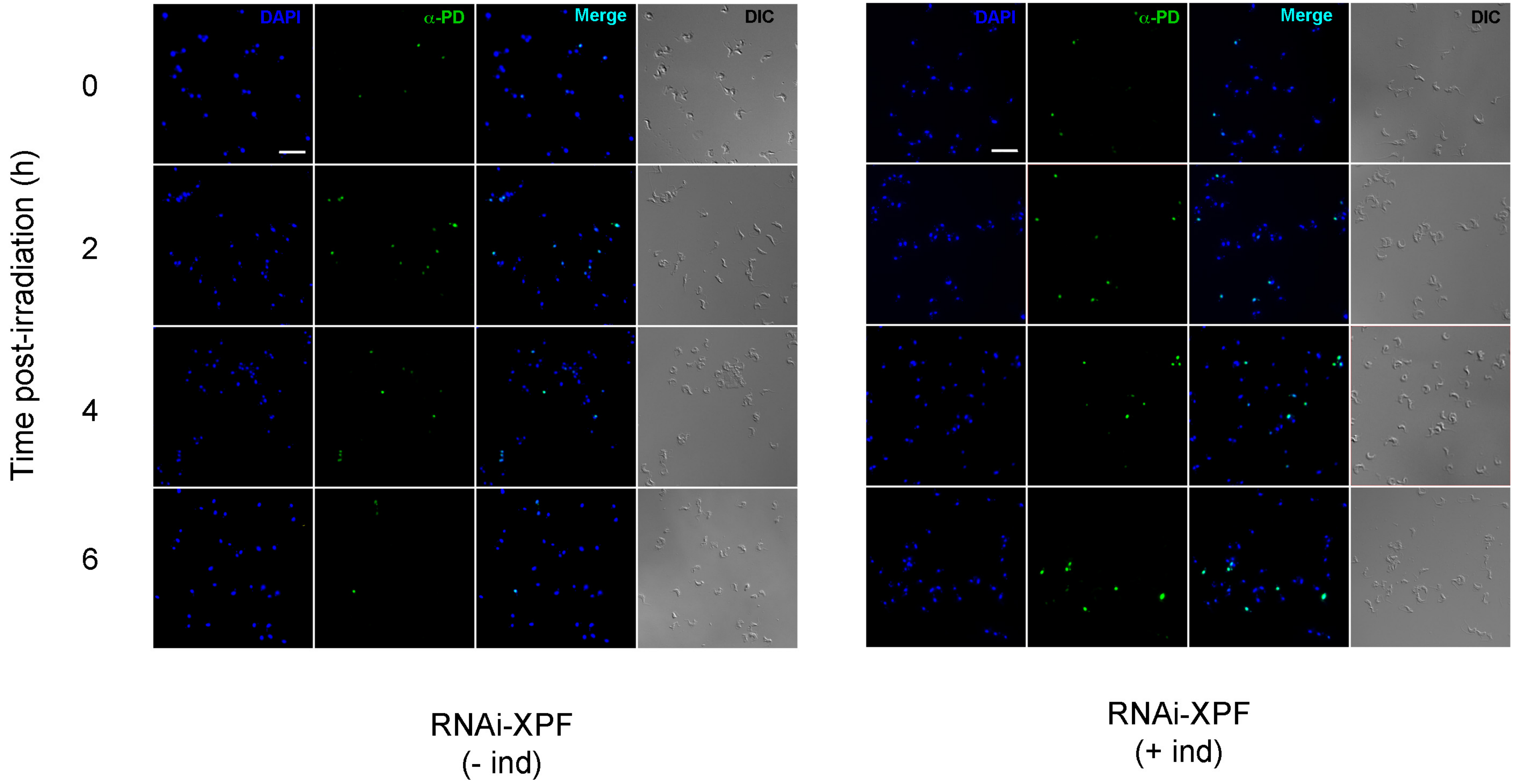

Figure S2. Detection of pyrimidine dimers (PD) by immunofluorescence microscopy. Representative image fields of *T. brucei* cells at different time points post-irradiation with 50 J/m² UVC. Images include DAPI staining to confirm that PD signal is restricted to DNA-containing compartments. These wider-field views were used to quantify the proportion of PD-positive cells across the population. Images shown are representative of three independent biological replicates.

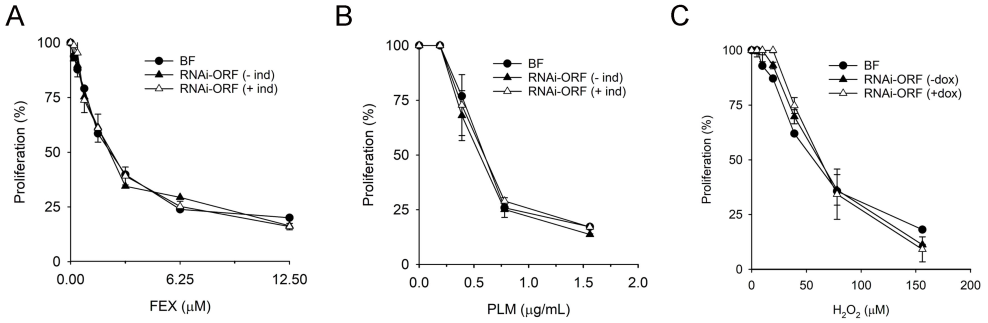

Figure S3. Proliferation assay of NER-defective and parental parasites exposed to genotoxic compounds. Log-phase parasites were exposed to increasing concentrations of (A) fexinidazole (FEX); (B) phleomycin (PLM); and (C) hydrogen peroxide (H_2O_2) for 24 h at 37 ºC. Cell growth was measured with resazurin as described in Methods. For each cell line, proliferation was calculated relative to cell growth in the absence of compound. Experiments were performed at least three times, in duplicate. Values are the mean (±SD).

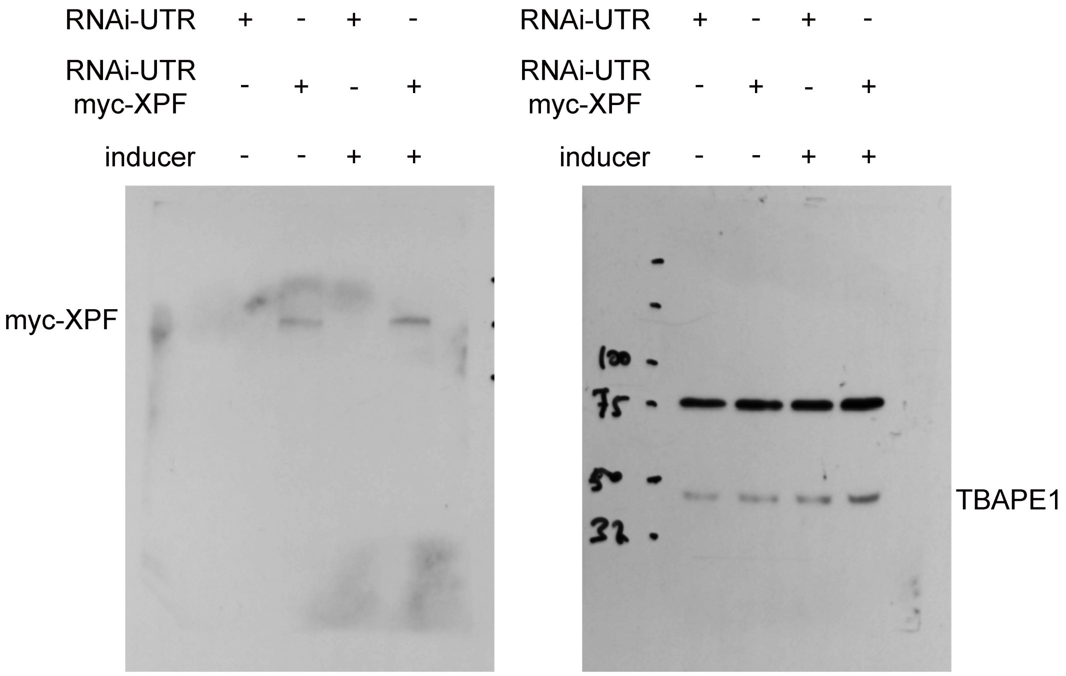

Figure S4. Full size images of western blots presented in Figure 6 of the manuscript. Western blot analysis of a transgenic cell line that expresses an inducible myc-tagged TbXPF and the RNAi targets the 3'-UTR of XPF (RNAi-UTR/myc-XPF). Myc-TbXPF was detected with anti-myc tag mouse monoclonal antibody (clone 4A6). The parental RNAi-UTR cell line was used a control. RNAi and myc-XPF expression was induced using 1 μg/mL of doxycycline. Right, TBAPE1 endogenous levels were used as loading control [35].

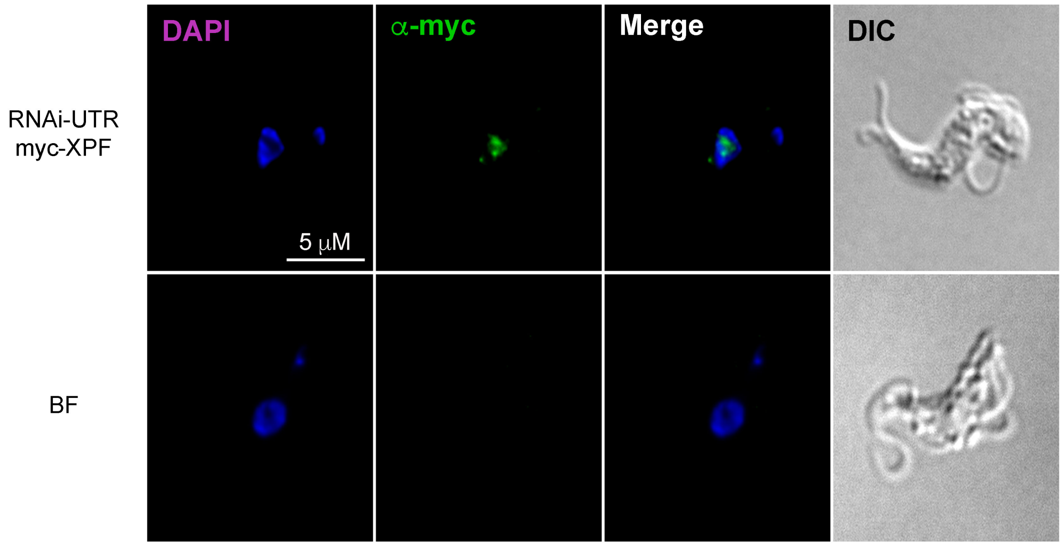

Figure S5. Detection of TbXPF in the nucleolar region using a second monoclonal anti-myc tag antibody (clone 4A6). Immunofluorescence microscopy images from parental (BF) and myc-expressing parasites (RNAi-UTR/myc-XPF) were obtained using an anti-myc tag mouse monoclonal antibody (clone 4A6) and Alexa Fluor 488 goat anti-mouse secondary antibody. Nuclear and kinetoplast DNA were stained with DAPI. Images were collected with an inverted Leica DMi8 microscope, 100x objective and LASX software.

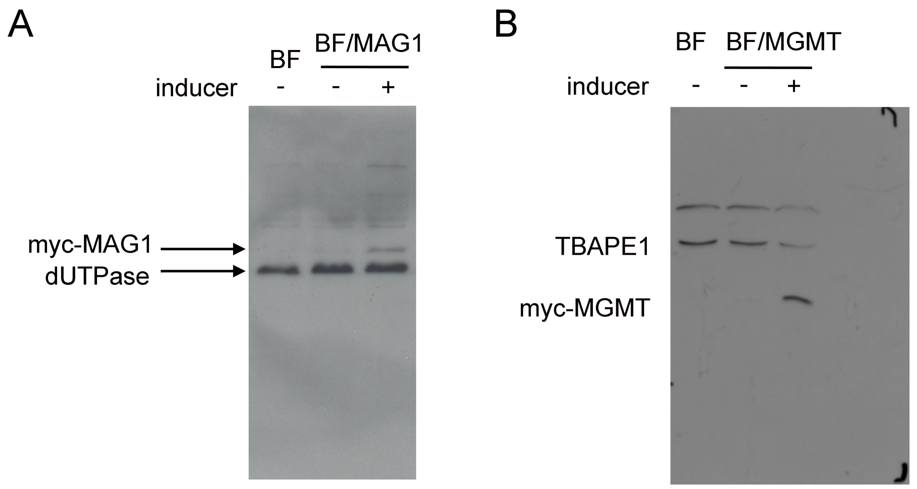

Figure S6. Full size images of western blots presented in Figure 7 of the manuscript. Western blots showing (A) MAG1 or (B) MGMT protein levels in whole cell extracts from 5 x 10^6 parasites. Samples and controls derived from the same experiment. Blots were generated through sequential incubations with the different antbodies (anti-myc, anti-dUTPase and anti-TBAPE1). Cell lines analyzed include: parental bloodstream form (BF), myc-tagged MAG1-expressing cells (BF/MAG1) and myc-tagged MGMT-expressing cells (BF/MGMT), in the absence (-) or presence (+) of the inducer (1 μg/mL doxycycline). As loading control, (A) an anti-dUTPase or (B) anti-TBAPE1 antibodies were used [35,60].

Table S1. List of oligonucleotides used in this study.

Sequence (5'>3')	Description	Use
AAA AAG TAA AAT TCA CAA GCT TGA TGG CAC AAG ATG TGT TAT C	Forward primer to amplify the ORF of XPF. Contains a complementary sequence to pGR19 and a HindIII site	Generation of an RNAi vector by In-fusion cloning
GAT GGC CGC TCT AGA ACT AGC TGC GGT CAA ACT CAA TAA G	Reverse primer to amplify the ORF of XPF. Contains a complementary sequence to the Stuffer fragment	Generation of an RNAi vector by In-fusion cloning
CTG GGG CGT GCA GGA CCA GCC GCT GCG GTC AAA CTC AAT AAG	Forward primer to amplify the ORF of XPF. Contains a complementary sequence to the Stuffer fragment.	Generation of an RNAi vector by In-fusion cloning
CAA CCC GGT GTT AGG ATC CGT TGA TGG CAC AAG ATG TGT TAT C	Reverse primer to amplify the ORF of XPF. Contains a complementary sequence to pGR19	Generation of an RNAi vector by In-fusion cloning
CTA GTT CTA GAG CGG CCA TC	Forward primer to amplify the Stuffer fragment of pGR19	Generation of an RNAi vector by In-fusion cloning
CGG CTG GTC CTG CAC GCC CCA G	Reverse primer to amplify the Stuffer fragment of pGR19	Generation of an RNAi vector by In-fusion cloning
AAA AAG TAA AAT TCA CAA GCT TAC TTG TGT TTG GTT GTG TTA G	Forward primer to amplify the 3'UTR of XPF. Contains a complementary sequence to pGR19 and a HindIII site	Generation of an RNAi vector by In-fusion cloning
GAT GGC CGC TCT AGA ACT AGC ATA CCT CCA CTC AGA GAA G	Reverse primer to amplify the 3'UTR of XPF. Contains a complementary sequence to the Stuffer fragment	Generation of an RNAi vector by In-fusion cloning
CTG GGG CGT GCA GGA CCA GCC GCA TAC CTC CAC TCA GAG AAG	Forward primer to amplify the 3'UTR of XPF. Contains a complementary sequence to the Stuffer fragment.	Generation of an RNAi vector by In-fusion cloning
CAA CCC GGT GTT AGG ATC CGT TAC TTG TGT TTG GTT GTG TTA G	Reverse primer to amplify the 3'-UTR of XPF. Contains a complementary sequence to pGR19.	Generation of an RNAi vector by In-fusion cloning
GAT GGT TGT TGT GTT TGG TC	Forward primer (qPCR)	Quantification of TbXPF mRNA in RNAi-ORF cells
CTG CTT CTC GGT ATC GTT GTC	Reverse primer (qPCR)	Quantification of TbXPF mRNA in RNAi-ORF cells
CAG CGT GTC ATG GCA CTT TG	Forward primer (qPCR)	Quantification of TbXPF mRNA in RNAi-UTR cells
CTACATATTGGGTCTAACAC	Reverse primer (qPCR)	Quantification of TbXPF mRNA in RNAi-UTR cells
CAT ATG CCA CAG ACT GTT AGC	Forward primer to amplify TbXPF. Contains an NdeI site for cloning into pGRV23	Expression of TbXPF in *T. brucei* cells
AGA TCT TTA TGT CTG AGT AGG TGG C	Reverse primer to amplify TbXPF. Contains a BglII site for cloning into pGRV23-myc	Expression of Myc-TbXPF in *T. brucei* cells
AGA TCT TTA TTT GTC GTC ATC GTC TTT GTA GTC TGT CTG AGT AGG TGG CAC	Reverse primer to amplify TbXPF. Includes a C-terminal FLAG tag. Contains a BglII site for cloning into pGRV23	Expression of TbXPF-Flag in *T. brucei* cells
GAC CAT ATG GAC AAG GAT TGT GAA ATG	Forward oligo to amplify MGMT. Contains an NdeI site for cloning into pGRV23-myc	Expression of myc-MGMT in *T. brucei* cells
AGT GGA TCC TCA GTT TCG GCC AGC AGG C	Reverse oligo to amplify MGMT. Contains a BamHI site for cloning into pGRV23-myc	Expression of myc-MGMT in *T. brucei* cells
AGG GCA ATT AAT ATG GAG GAG CAG AAG CTG ATC	Forward oligo to amplify MAG1. Contains an AseI site for cloning into pGRV23-myc	Expression of myc-MAG1 in *T. brucei* cells
AAG CTT GGA TCC TTA GGA TTT CAC GAA ATT TTC	Reverse oligo to amplify MAG1. Contains a BamHI site for cloning into pGRV23-myc	Expression of myc-MAG1 in *T. brucei* cells
